# Ethnic Differences in Weight Category Transition and Body Mass Index Trajectories from Adolescence to Young Adulthood in Israel

**DOI:** 10.3390/children12121625

**Published:** 2025-11-30

**Authors:** Yulia Treister-Goltzman, Dan Nemet, Idan Menashe

**Affiliations:** 1 Department of Family Medicine and Siaal Research Center for Family Practice and Primary Care, The Haim Doron Division of Community Health, Faculty of Health Sciences, Ben-Gurion University of the Negev, Beer-Sheva 84161, Israel; 2 Clalit Health Services, Southern District, Beer-Sheva 84161, Israel; 3 Child Health and Sports Center, Meir Medical Center, Kfar-Saba 4428164, Israel; 4 Gray School of Medicine, Tel Aviv University, Tel Aviv 6139001, Israel; 5 Department of Epidemiology, Biostatistics, and Community Health Sciences, Faculty of Health Sciences, Ben-Gurion University of the Negev, Beer-Sheva 84105, Israel

**Keywords:** BMI trajectories, adolescent weight category, ethnicity, multilevel modeling, young adulthood, obesity

## Abstract

**Highlights:**

**What are the main findings?**

- Many adolescents in Israel have an increasing BMI trajectory and move to a higher weight category in adulthood
- This trend is especially prominent for Arab adolescents

**What is the implication of the main finding?**

- Groups with an especially high risk for BMI increase: Arab adolescents, adolescents from low SES, and females
- Initiatives for obesity prevention, especially in risk groups, need to be intensified

**Abstract:**

Objective: To assess ethnic differences in the transition of weight categories and in BMI trajectories from adolescence to young adulthood between Jewish and Arab populations. Methods: A retrospective cohort study, based on the centralized computerized database of ‘Clalit Health Services’, the major health service organization that insures 52% of the Israeli population. The study population consisted of 99,741 adolescents born from 1988 to 1992 who had at least one BMI measurement in the exposure (ages 17–19 years) and follow-up (age 20 to <30 years) periods. We examined differences in weight categories in young adulthood (ages 20 to <30 years), by ethnic group, and assessed whether ethnicity moderated BMI trajectories from 20 to <30 years of age. Multilevel models were applied to examine BMI trajectories. Results: A higher percentage of Arab than Jewish adolescents from the ‘underweight’, ‘normal’, and ‘overweight’ categories moved to one of the higher weight categories in young adulthood (*p* < 0.001 for all). In the ‘underweight’, ‘normal’, ‘overweight’, and ‘obese’ weight categories, the increase in BMI with increasing age was lower for Jews (*p* < 0.001 for all). Conclusions: Many adolescents, even if not obese, have increasing BMI trajectories at ages 17–19 and move to a higher weight category in adulthood. Arab adolescents constitute a risk group for rapid BMI increase as they transition to adulthood. The present study could contribute to a better understanding of BMI dynamics and, in turn, to the development of more effective public health and policy interventions.

## 1. Introduction

Adolescent obesity is an important risk factor for physical and psychiatric morbidity, even for young adults [1,2,3,4,5,6,7]. Body mass index (BMI) in adolescence is strongly associated with adult BMI, even with BMI in offspring and intergenerational obesity [8,9]. A recent in-depth meta-analysis demonstrated that the age-standardized prevalence of obesity in children and adolescents increased in recent decades in more than 95% of the countries in the world. In most countries, obesity has more than doubled [10]. The median prevalence of adolescent obesity was reported to be more than 31% in some of the Oceania islands and more than 20% in North American countries [11]. Both period prevalence (increasing obesity in recent years) and cohort prevalence (increasing obesity in recent birth cohorts) play a role in the increasing burden of obesity [12,13], which poses a major threat to current and future generations.

Given these trends and previously observed ethnic differences in growth and weight patterns, we hypothesized that BMI increases from adolescence to young adulthood would be greater among Arabs than among Jews. Different BMI trajectories among different ethnic groups in the same geographical area have been described as early as childhood in multiple countries [14,15,16,17,18].

The population of Israel in 2024 is estimated to be 10 million persons. The Jewish population comprises 74.6% of the total, 21.4% are Arabs, and 4% are others [19]. Higher mortality rates for Arabs in Israel are mostly for diabetes, respiratory diseases, and heart disease [20], all of which are etiologically related to obesity. The rates of both pediatric and adult obesity have been increasing in Israel in recent years [21,22]. According to the latest survey of the Israeli Ministry of Health, 56% of the general population of Israel is overweight and obese [22]. The rate is higher among Arabs (61.3%) compared to Jews (54.8%). Socioeconomic disparities shape access to healthy foods, physical activity, and health knowledge, contributing to obesity risk, which in turn reinforces inequalities in productivity and social opportunities, particularly among marginalized ethnic groups. In our conceptual framework, ethnicity serves as a moderator of BMI trajectories from adolescence to young adulthood, while SES is included as a covariate to account for confounding. To date, no study has examined ethnic differences in BMI trajectories in Israel, underscoring the importance of this investigation for equitable public health interventions.

The main goals of the present study were to compare ethnic differences in weight category transition and in the longitudinal changes in BMI trajectories in each adolescent weight category from adolescence to young adulthood between the Jewish and Arab populations. Another goal was to assess sex differences in these trajectories in each ethnic group.

## 2. Methods

This was a retrospective cohort study, based on the centralized computerized database of ‘Clalit Health Services’ (CHS), the major health service organization that insures 52% of the Israeli population. As an integrated health care provider and insurer, CHS has a centralized database containing the clinical, financial, and administrative data of all insured members since 2000. All of these data are linked through a unique identifier and are geo-coded with the patient’s clinic and home address and aligned with area-level statistics. Hence, all the measurements used in the present study are standardized. It should be noted; however,, however, that this data source has a potential limitation due to the lack of individual-level information on factors such as lifestyle, physical activity, or diet. The study population consisted of adolescents born in 1988–1992 who had at least one measurement of weight and height at ages 17–19 years and at ages 20–30 years. Participants with major chromosomal anomalies and/or intellectual disabilities were excluded from the cohort. The study period was from 1 January 2007 to 31 December 2022. It was divided into an exposure period from 1 January 2007 to 31 December 2011 (when participants were 17–19 years of age) and a follow-up period from 1 January 2008 to 31 December 2022 (when the participants were 20 to less than 30 years of age). The data collected included socio-demographic data: age, sex, ethnic sector, district of residence, socio-economic status (SES, defined in the “Clalit” computerized database as low, middle and high by zip code), dates of insurance termination in CHS, all height (cm), weight (kg), and BMI measurements, and any recorded diagnosis of major chromosomal anomalies and/or intellectual disabilities. Additionally, the total number of insured adolescents aged 17–19 years during the study period was obtained to assess the rate of missing data.

### 2.1. Definitions of Variables

#### 2.1.1. Main Exposure Variable

BMI was defined as weight in kilograms divided by height squared in meters. Adolescent weight categories were defined as percentiles determined by the U.S. Center for Disease Control and Prevention (CDC), which were validated for Israeli adolescents as ‘underweight’ (BMI < 5th percentile), ‘normal weight’ (5th–84.9th percentile), ‘overweight’ (85th–94.9th percentile), ‘obesity’ (≥95th percentile, but not including ‘Class 2’ and ‘Class 3 obesity’). ‘Class 2 obesity’ was diagnosed if BMI reached ≥120% to <140% of the 95th percentile or BMI was ≥35 to <40 kg/m^2^. ‘Class 3 obesity’ was diagnosed if BMI was ≥140% of the 95th percentile or BMI ≥ 40 kg/m^2^ [23,24]. BMI percentiles are calculated automatically in electronic health records.

#### 2.1.2. Main Outcome Variable

The primary outcome for the first goal was the change in BMI category between the exposure and the follow-up periods. The outcome BMI value was defined as the last available BMI measurement during the follow-up period. Outcome BMI values were used to classify individuals as: underweight (<18.5 kg/m^2^), healthy weight (18.5 to <25 kg/m^2^), overweight (25 to <30 kg/m^2^), obesity (30 to <35 kg/m^2^), class 2 obesity (35 to <40 kg/m^2^) and class 3 obesity (≥40 kg/m^2^). The outcome in the model-based analysis was BMI trajectories during the follow-up period (ages 20 to <30 years). The analysis was stratified by baseline adolescent weight category.

Adolescent BMI categories were defined using CDC age- and sex-specific percentiles, which account for physiological growth during puberty. In young adulthood, BMI categories were defined using WHO fixed cut-points that represent absolute risk thresholds. This approach ensured age-appropriate and comparable classification across the transition from adolescence to adulthood.

#### 2.1.3. Other Variables Definitions

Major chromosomal and other congenital anomalies were defined by the International Classification of Diseases, Ninth Revision (ICD-9) codes of 758.0, 758.1, 758.2, 758.3, 758.5, 758.6, 758.7, 758.8, 758.9, 759.5, 759.6, 759.7, 759.8, 759.9, and moderate to severe intellectual disabilities by ICD-9 codes 318 and 319. The allocation into one of these ethnic groups was based on the city of residence and the patient’s clinic location. Adolescents who received care in community clinics located in Arab localities, but resided in a Jewish city, were classified as Arab ethnicity. The classification of the two main ethnic groups in Israel reflects a combination of genetic, cultural, religious, and linguistic characteristics.

### 2.2. Statistical Analyses

Data cleaning was performed, and outlying BMI values were deleted. We used a clinical approach to the definition of outliers. Data points that deviated significantly from the rest of the dataset due to obvious errors in data collection or recording were defined as outliers, so-called illegitimate outliers (BMI ≤ 10 and BMI ≥ 60). The study population was completed after exclusion of patients with major chromosomal abnormalities and intellectual disabilities. We compared the basic socio-demographic characteristics of the adolescents with and without BMI measurements to assess for possible selection bias. For adolescents with several weight and height measurements at ages 17–19, we chose the earliest BMI measurement for further analyses. We characterized the baseline features of the study population using descriptive statistics.

The study population was stratified by weight category, and two separate analyses were performed for each category. The first analysis examined differences in weight categories in young adulthood (ages 20 to <30 years), by ethnic group. For this analysis, the last BMI measurement in young adulthood was taken for each participant. We used the χ^2^ test to test for differences in adult weight categories between the ethnic groups.

**Figure A1:**
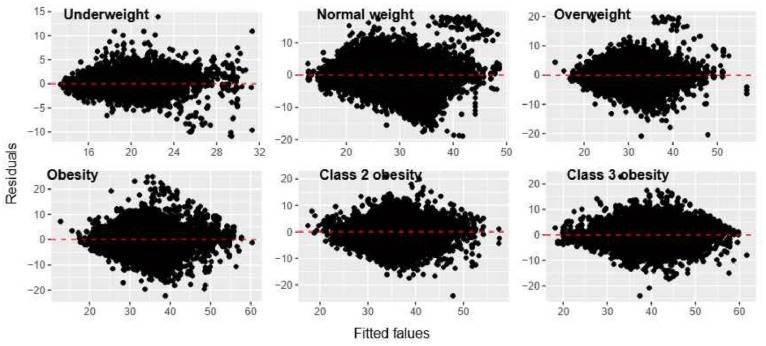
Residuals vs. fitted plots of multilevel models estimating BMI trajectory.

**Figure A2:**
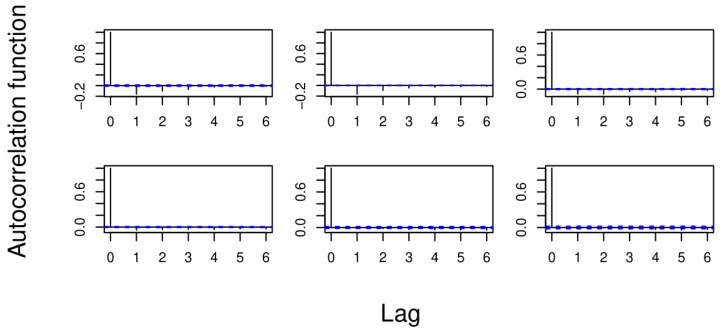
Autocorrelation function examining independence of residuals. Upper row from left to right: ‘underweight’, ‘normal’, ‘overweight’ weight categories. Lower row, from left to right: ‘obesity’, ‘Class 2 obesity’, and ‘Class 3 obesity’ weight categories.

In the second analysis, we examined whether ethnicity moderated the BMI trajectory from 20 to <30 years of age in each adolescent weight group. Multilevel linear regression models for repeated measurements [25] were applied to examine the trajectories of BMI. Briefly, the multilevel models estimated the mean trajectories of the outcome while accounting for repeated (non-independent) measurements within individuals and differences in the number and timing of measurements between individuals (using all available data from all eligible participants under a missing at random assumption). We tested the assumption of linearity by examining residual plots (residuals vs. fitted plots are demonstrated in Figure A1) and the independence of residuals by checking for autocorrelation (Figure A2). Both tests confirmed linearity, enabling us to build mixed linear regression models with constant variance structure. In our analysis, repeat measurements of BMI were nested within each participant during the follow-up period (i.e., each year from 20 to <30 years of age). Trajectories were estimated using linear models in a multilevel framework (measurements within individuals (level 1) and between individuals (level 2). The polynomial quadratic age parameter (age of measurement ^2^) for the non-linear shape of trajectories was not significant and hence was not included. We explored whether BMI trajectories differed by ethnic group by fitting an interaction term between age in young adulthood and ethnic group. Four nested models were built for each adolescent weight category: Four nested models were constructed for each adolescent weight category: (a) Null, partitioning BMI variance; (b) Model 1, adult age-BMI association; (c) Model 2, additionally adjusted for ethnicity, sex, and SES; (d) Model 3, including an interaction between adult age and ethnic group. Random effects for slopes were included in all models.

The general formula for the models can be presented as:

Y_ij_ = b_0_ + b_1_X_1ij_… + b_e_X_pij_ + u_1_X_1ij_… + u_e_X_pij_ + e_1ij,_

where Y_ij_ is a dependent variable (BMI) for individual i at time j, b_0_ is the overall intercept, b_1_…b_p_ are the fixed-effect coefficients for the independent variables, X_1ij_ … X_pij_ are the values of the independent variables for individual i at time j, u_1i_ …, u_pi_ are the random slopes for individual i, representing the deviation of individual i’s slopes from the overall slopes, and e_ij_: is the random error term for individual i at time j. In the Model 3, an interaction term was added.

For figure illustrations and a more intuitive interpretation, we presented Model 3 estimates graphically to show BMI trajectories by ethnic group for each weight category.

To examine potential non-linear patterns, including the possibility of a plateau or reversal in the late 20s, we tested spline models with knots placed at each year between ages 24 and 27. A knot at age 25 was the only statistically significant one, and therefore, spline models with a knot at age 25 were fitted for each weight category. Goodness-of-fit statistics for the spline models were compared with those of the corresponding linear models.

To demonstrate sex differences in BMI trajectories by ethnic group in each weight category, we graphically presented the trajectories predicted by the models with a triple interaction term between age, ethnic group, and sex, adjusted for SES.

The ‘missing at random’ assumption was tested via inverse probability weighting, and further sensitivity analyses are detailed in Table A1.

Statistical analyses were performed using the lme4 package in R (version 4.3.3) with maximum likelihood estimation. Model fits were assessed using Akaike information criterion, Bayesian information, and deviance.

Patients’ data were deidentified using the MDClone platform, and all statistical analyses were carried out with the Virtual Desktop Infrastructure to ensure patient anonymity.

Statistical significance was set at two-sided *p* < 0.05.

## 3. Results

**Figure 1:**
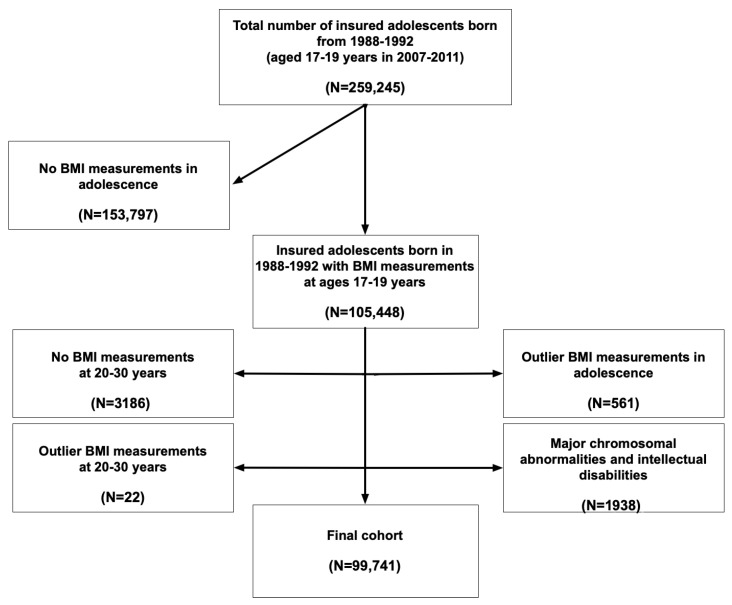
Flowchart of the study cohort selection.

A flowchart of the study selection process is presented in Figure 1. Of the 259,245 adolescents insured by CHS who reached ages 17–19 between 2007 and 2011, 105,448 had recorded weight and height measurements. Comparisons between participants with and without BMI measurements (or with outlying BMI values) indicated that those with measurements included fewer males, fewer Jews, and a higher proportion of individuals in lower SES categories (*p* < 0.001 for all comparisons) (Table A2). Additionally, fewer participants with measurements resided in Jerusalem compared with those without (*p* < 0.001). The final sample consisted of 99,741 participants.

Table A3 presents the sociodemographic characteristics of the cohort of adolescents by weight and ethnic group. Sex differences reached statistical significance only in the ‘normal’ weight category (46.2% vs. 45.1% males, *p* < 0.01 in the Arab and Jewish groups, respectively). The difference in SES was significant (*p* < 0.001) in all weight categories, with many more Jews (11.9–19.2% in different weight categories) than Arabs (less than 1%) belonging to the high SES. Many more Jews than Arabs resided in the more centrally located districts of Israel, and many more Arabs in the Northern, Haifa, and Jerusalem districts (*p* < 0.001). Although the baseline BMI measurements were carried out between 17 and 19 years among all adolescents, this age was significantly lower among Jewish than among Arab adolescents (17.8–18.1 vs. 18.1–18.3 years, respectively, in the different weight categories *p* < 0.001). It is noteworthy that although statistically significant, this difference did not exceed 0.3 years. The mean BMI (SD) was significantly higher among Arabs than Jews in most weight categories at *p* < 0.001, although these differences did not exceed 0.5 kg/m^2^. The baseline differences justified adjustments in multivariable models.

The details of BMI measurements during the follow-up period, by ethnic group, are provided in Table A4. Less than half of the participants were Jews (47.2%), but they contributed 50.5% of the BMI measurements (*p* < 0.001). More than half of the participants in both groups had 2–4 measurements. The mean age of measurements was significantly lower among Arab participants than among Jewish participants, as was the last age of BMI measurement (23.5 (2.8) vs. 24.1 (2.9), and 25.9 (2.5) vs. 26.4 (2.6), respectively, *p* < 0.001 for both comparisons).

### 3.1. Change in Weight Category from Adolescence to Young Adulthood in Arabs and Jews

**Figure 2:**
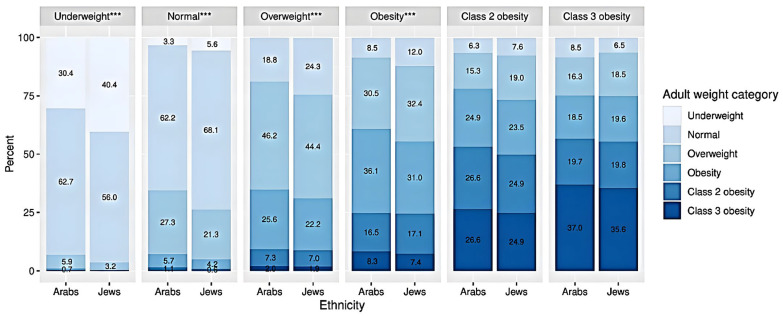
Change in BMI category from adolescence to young adulthood. Underweight = BMI < 5th percentile, normal weight = BMI 5th–84.9th percentile, overweight = BMI 85th = 94.9th percentile, obesity = BMI ≥ 95th percentile, not including class 2 and class 3 obesity, class 2 obesity = BMI ≥ 120% to <140% of the 95th percentile or BMI ≥ 35 to <40 kg/m^2^, class 3 obesity = BMI ≥ 140% of the 95th percentile or BMI ≥ 40 kg/m^2^. *** The differences are significant at *p* < 0.001.

A higher percentage of Arab than Jewish adolescents in the ‘underweight’, ‘normal’, and ‘overweight’ categories moved to a higher weight category in young adulthood (69.6% vs. 59.6%, 34.5% vs. 26.3%, and 34.9% vs. 31.1%, respectively) (Figure 2). A higher percentage of Jewish than Arab adolescents in the ‘normal’, ‘overweight’, and ‘obesity’ categories moved to a lower weight category. The χ^2^ test for the comparison of proportions was significant at *p* < 0.001 for ‘underweight’, ’normal’, ‘overweight’, and ‘obesity’ adolescent weight categories. In the extreme obesity categories (‘class 2’ and ‘class 3 obesity’), around half of the Jewish adolescents and close to half of the Arab adolescents moved to a lower weight category. The rest remained extremely obese in young adulthood without any significant differences between the ethnic groups.

Table A5 summarizes the number of adolescents in the ‘underweight,’ ‘normal,’ ‘overweight,’ and ‘obesity’ categories who transitioned to a higher weight category in young adulthood.

### 3.2. BMI Trajectories in Young Adulthood by Ethnic Group

**Figure 3:**
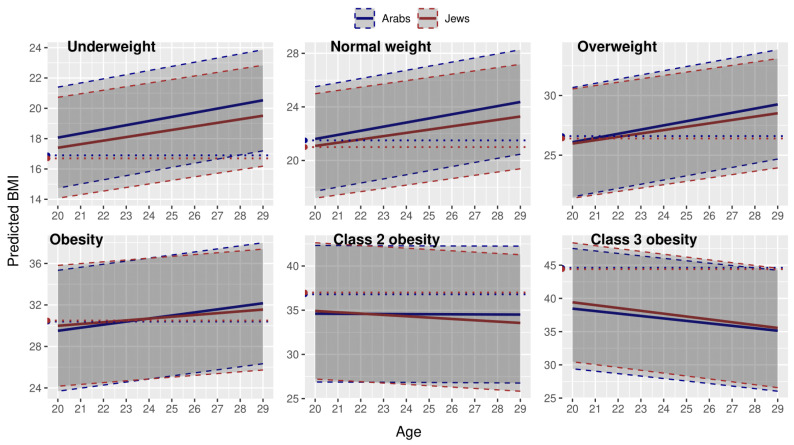
Trajectories of BMI change in young adulthood in adolescents from different weight categories, by ethnic group. Horizontal dotted lines = mean BMI values in 17–19 years. Dashed lines = 95% confidence intervals. Predicted by multilevel modeling, adjusted for sex and SES.

The predicted BMI trajectories of the fully adjusted models (Model 3) for each ethnic group are displayed in Figure 3. The detailed results of the Null models and of Models 1–3 are provided in Table A6. For adolescents in the ‘underweight’, ‘normal’, ‘overweight’, and ‘obese’ categories, the increase in age was associated with an increase in BMI (*p* < 0.001 for all). In adolescents in the ‘class 2 obesity’ category, there was no significant change in BMI with an increase in age, and in those in the ‘class 3 obesity’ category, there was a decrease, *p* < 0.001. Jewish ethnicity was associated with lower BMI in the ‘underweight’ and ‘normal’ weight categories (both at *p* < 0.001), but with a higher BMI (*p* < 0.001) in the ‘obesity’ category. This association was not significant in the other weight categories. The trajectories of change in BMI were different by ethnic group in all but the ‘class 3 obesity’ category. In the ‘underweight’, ‘normal’, ‘overweight’, and ‘obesity’ categories, the increase in BMI with increasing age was lower for Jews (the Age × Ethnicity (Jews) interaction term), at *p* < 0.001 for all. In the ‘class 2 obesity’ category, the trajectory of the BMI decrease with age was almost flat for Arabs, but much steeper for Jews. It is particularly noteworthy that the overlapping confidence intervals of the trajectories of the two ethnic groups do not imply an absence of effect modification [26]. Indeed, the cumulative difference (95% CI) in predicted BMI between two ethnic groups after 10 years of follow-up was non-significant in the ‘class 3 obesity’ category only (Table A7) and varied between 0.61 (0.30–0.92) in the ‘obesity’ category and 1.09 (1.00–1.17) in the ‘normal’ weight category.

Additional findings of interest from the models were a positive association of male sex with BMI in the ‘underweight’ and ‘normal’ weight categories. In contrast, there was a negative association in the ‘overweight’ and ‘obesity’ categories (*p* < 0.001 for all). Middle and lower, compared to high SES, were associated with an increase in BMI in all, except the ‘underweight’ and ‘class 3 obesity’ categories, in which the association between SES and BMI was not significant.

**Figure 4:**
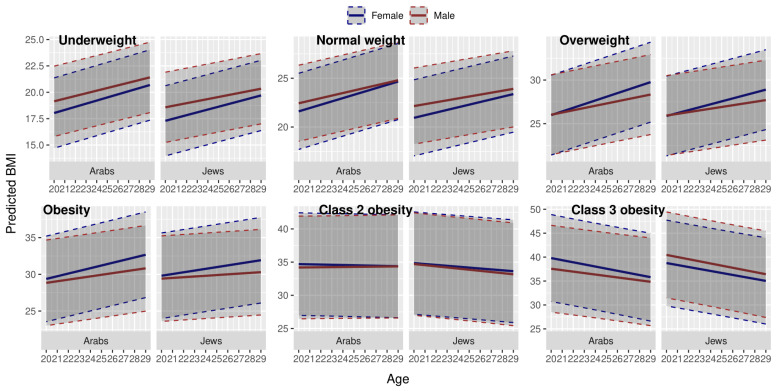
Trajectories of BMI change in young adulthood in adolescents from different weight categories, by sex and ethnic group. Dashed lines = 95% confidence intervals. Predicted by multilevel modeling, adjusted for SES.

Figure 4 depicts sex differences in BMI trajectories in Jews and Arabs in different weight categories. Females had higher BMI values throughout the trajectory in all excessive weight categories (from ‘overweight’ to ‘class 3 obesity’), with the single exception of Jewish females in the ‘class 3 obesity’ category, who had lower BMI values compared to males. Females had a more rapid BMI increase in the ‘normal’, ‘overweight’, and ‘obesity’ categories in both ethnic groups. In the ‘class 2 obesity’ category, Arab females had a downward trajectory, while in males, the trajectory was almost constant.

### 3.3. Sensitivity Analyses

Since significant differences were observed between individuals with available measurements and those without (fewer males, fewer Jews, lower SES in the group with measurements, *p* < 0.001 for all), the ‘missing at random’ assumption was tested by sensitivity analyses: (a) inverse probability weighting, assigning weights to study participants based on the inverse probability of their selection into the study and creating a pseudo-population that better represents the target population. These weights were derived from a logistic regression model that predicted the probability of being selected based on ethnicity, SES and gender (Table A8); (b) a sensitivity analysis limited to the first four measurement points per person since Jews contributed more measurements per person (5.0 vs. 4.4, Table A9) Both analyses yielded estimates almost identical to those of the main analysis.

The first research question was based on the last available BMI in adulthood, which exposed the results to bias due to observation duration and attrition rate (different ‘last age of measurement’: 26.4 vs. 25.9 years). When the outcome was defined as an averaged BMI from a fixed age window (24–26 years), the results of this part of the analysis persisted (Table A10).

**Figure A3:**
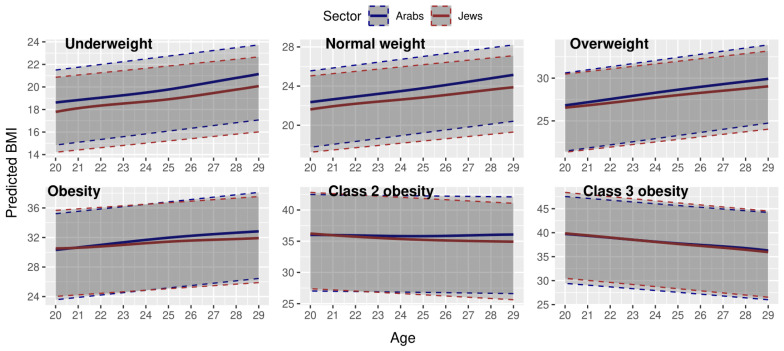
Trajectories of BMI change in young adulthood in adolescents from different weight categories, by ethnic group, predicted by multilevel linear spline modeling. Dashed lines = 95% confidence intervals.

Linear spline models were built to test the possibility of plateau/reversal weight trajectories in the late 20s. A knot at 25 years was the only statistically significant result, so spline models were evaluated with the knot at 25 years for each weight category. Although statistically significant, the incorporation of the spline did not improve the models’ performance, and BMI trajectories were visually almost identical to those of the primary analysis. The spline models’ estimates, the models’ fit assessment, and a graphic presentation of the models are shown in Appendix A, Table A11, and Figure A3.

We included district of residence as a fixed variable in the multilevel linear models without improvement in the model fit parameters (Table A12).

**Figure A4:**
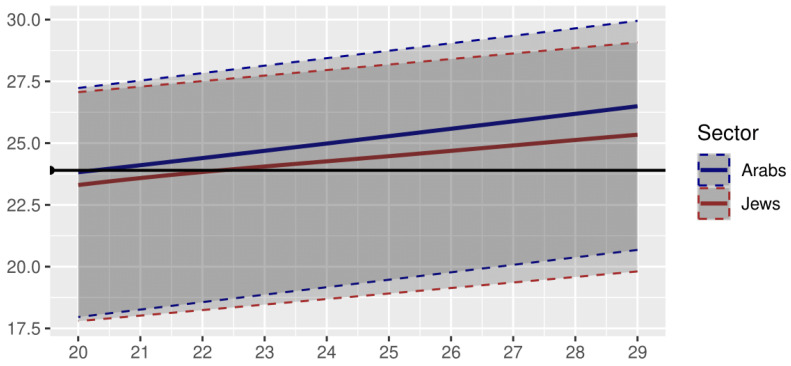
Trajectories of BMI change in young adulthood in the whole study cohort, by ethnic group. Horizontal solid line = mean BMI in ages 17–19 years, dashed lines = 95% confidence intervals. Predicted by multilevel modeling, adjusted for sex and SES.

Lastly, the use of CDC thresholds for weight categories among adolescents, but WHO thresholds for adults, could create artifacts of categorical transitions around 18.5/25/30. We performed a sensitivity analysis with BMI as a continuous variable for the whole population of participants, by ethnicity (Table A13, Figure A4). This analysis confirmed the more gradual increase in BMI with increasing age for Jews.

## 4. Discussion

The first important finding of the present study is the consistent increase in BMI throughout young adulthood until the age of 30 years in Israeli adolescents in most weight categories. A substantial proportion of the adolescents moved to higher weight categories. However, adolescents from the ‘class 3 obesity’ category showed a decline in BMI in young adulthood, and most of them moved to lower weight categories. Only around 20% and 10% of participants from the ‘overweight’ and ‘obese’ weight categories, respectively, transitioned to the ‘normal’ weight category; fewer than that in the extreme ‘class 2’ and ‘class 3 obesity’ categories. A large study from the US, which examined a 1979–1980 birth cohort, also showed that most participants transitioned to higher BMI categories [27]. Its findings were even more negative, and only 8% of the participants from the ‘overweight’ and 2% from the ‘obese’ adolescent weight category moved to the ‘normal’ weight category in young adulthood.

Our study aimed at examining ethnic differences in the trajectories of BMI change, and such differences were indeed observed in all but the ‘class 3 obesity’ category. In the Jewish ethnic group, the trajectory of BMI increase in young adulthood was more moderate, and the trajectory of BMI change in the ‘class 2 obesity’ category in young adulthood declined in this ethnic group only. It should be noted that the cumulative difference in predicted BMI between the two ethnic groups, though statistically significant, was quantitatively and clinically moderate and reached, at most, 1.09 BMI points in the ‘normal’ weight category. With a mean male height in Israelis of 174.5 cm [28], this BMI difference corresponds to approximately a 3.3 kg difference in weight.

Nevertheless, the high prevalence of obesity suggests that even modest differences may translate into substantial public health significance. Studies have shown that each 1 kg/m^2^ increase in BMI is associated with an 8.4% higher risk of incident diabetes mellitus, a 4.2% higher risk of developing hypertension, and a 5.1% higher risk of left ventricular hypertrophy [29]. In addition, incremental increases in BMI are linked to elevated risks of coronary heart disease, heart failure, atrial fibrillation, and multiple other cardiovascular outcomes [30].

Steeper increases in BMI trajectories over the course of life were demonstrated previously for Black and Hispanic, compared to White, in the United States [31,32], Black Caribbean, compared to White, in the UK [33], and for Malay and Indian, compared to Chinese, in Malaysia [18]. Even among minority subgroups within the same country, BMI trajectories may vary in different directions; for example, Hispanics in the United States exhibit faster BMI increases, whereas Asians show slower increases compared with Whites [34]. Furthermore, several studies have shown that racial and ethnic disparities in BMI trajectories are already well established in early childhood [35,36] and that socioeconomic status is differentially associated with BMI trajectories across ethnic groups [36].

Previous studies that assessed the trajectories of BMI change usually assessed them in the whole study population. The novelty of the present study lies in its examination of these trajectories separately for each adolescent weight category to identify unique patterns of BMI change. Thus, it became possible to detect, for instance, an extremely rapid increase in BMI among adolescents in the ‘normal’ and ‘overweight’ categories, or a downward BMI trajectory for adolescents in the extreme ‘class 3 obesity’ category. Moreover, it facilitated the recognition of distinct ethnic patterns, as, for instance, the steeper increase in BMI in Arab adolescents in the ‘underweight’, ‘normal’, ‘overweight’, and ‘obese’ categories, with an especially large discrepancy in the ‘obese’ category, or the declining BMI trajectory for Jewish adolescents from the ‘class 2 obesity’ category only.

Low and middle SES, as compared to high SES, were found to be a risk factor for an increase in BMI in our study. In contrast, several other studies demonstrated a higher velocity of BMI increase in adolescents from high SES [37,38], though the findings from one study [39] were in line with ours. The lack of association between SES and BMI among adolescents with low weight was also found in another study [18]. Similar sex discrepancies, with a more rapid BMI increase in females than in males, in most weight categories, were demonstrated in two studies [27,40]. Hormonal factors, pregnancies, and behavioral differences may contribute to accelerated BMI growth in females [41]. Indeed, a large meta-analysis demonstrated that higher parity is associated with an elevated BMI prior to subsequent pregnancies [42]. Pregnancies in early adulthood may have a particularly pronounced impact on observed gender differences in BMI, given that fertility rates in Israel are relatively high, averaging approximately three children per woman, and are similar across the two ethnic groups [43].

Although ethnicity was modeled as a moderator, the observed differences are likely shaped by intersecting sociocultural, behavioral, and environmental-structural mechanisms. Sociocultural factors among Arab populations include the transition from traditional diets to energy-dense, westernized foods, norms favoring large family meals, and gender-specific constraints on leisure-time physical activity. Behavioral mechanisms involve lower participation in structured physical activity, higher consumption of fried and processed foods, and disparities in health literacy. Environmental and structural conditions, such as socioeconomic disadvantage, limited access to recreational infrastructure, and food environments with greater availability of fast-food outlets and fewer affordable healthy options, further reinforce these patterns [44,45]. Indeed, ECHO Program cohort analyses, drawing on data from 54 cohorts [46] and 55 cohorts [47] spanning birth through adolescence, suggest that early-life neighborhood resources and the food environment have long-term implications for weight gain. These associations remained significant even after adjusting for family sociodemographic characteristics. There are notable ethnic disparities in preventive health care utilization, including lower rates of dietitian consultation within the Arab population [48].

While there are national estimates of the prevalence of obesity, less is known about its dynamics in the transition from adolescence to adulthood or the ethnic differences in these dynamics. Obesity has long-term social, economic, and health implications, and a better understanding of its dynamics can help us understand how these implications play out. Our study is an effort to explore such dynamics in the critical period of adolescence and young adulthood in a nationally representative longitudinal study.

### Study Strengths and Limitations

Strengths. The main strength of this study is that it was based on a nationwide, representative, large, and reliable database, with many observations. Another strength is that the weight and height measurements and diagnosis recordings were performed by health care professionals, rather than being self-reported. Weight categories were defined separately for adolescents and adults, according to age-appropriate CDC definitions, compatible with real-life weight category definitions at each age in Israel and many other countries. The allocation to the ‘class 2’ and ‘class 3’ obesity categories made it possible to describe the unique patterns of BMI change in these obesity categories. This enabled us, for instance, to detect the flat BMI trajectory in the ‘class 2 obesity’ category, and the downward trajectory in the ‘class 3 obesity’ category, which were radically different from the trajectories in the other weight categories.

Limitations

Missing data bias and unequal measurement frequency. The main limitation of our study is basing the definition of exposure on BMI from medical records. A substantial proportion of participants did not have measurements during adolescence, and some lacked measurements during adulthood, which may introduce selection or survivor bias, as individuals who remained in the cohort may differ from those without measurements in terms of health status or mobility. Such bias could lead to either underestimation or overestimation of BMI trajectories. The recommendation to measure height and weight for BMI for all adolescents aged 14–19 years, and adults aged 20–30 years has been an integral part of the Israel National program for quality indicators in the community since 2007. Still, missing BMI measurements during adolescence and an imbalance in the number and time of measurements in the two ethnic sectors (Jews contributed more measurements during adulthood) could have led to selection bias.

Use of CDC thresholds for weight categories among adolescents and WHO thresholds for adults could create artifacts of categorical transitions around 18.5/25/30. On the other hand, statistical attempts to neutralize these constraints showed results that were consistent with the main analysis.

Residual confounding. There is still a high risk of ecological fallacy, as multiple individual-level factors, such as parental education, religiosity, migration status, physical activity, diet, smoking, urban/rural location, and personal psychological characteristics, were not included in the analyses. In the group of women aged 20 to <30 years, postpartum and pregnancy could affect BMI. We did not include pregnancy/birth variables, which could explain the faster BMI increase in women, demonstrated in our paper, at least in part.

BMI has limitations as a useful measure of obesity; its utility differs in different ethnicities, and it cannot distinguish between muscle and fat tissue. At the same time, BMI is still the most widely used measure in population-based epidemiological studies and has a good correlation with body fat percentage.

The specific ethnic nature of the study population limits its generalizability, but the goal of our study is to compare the impact of weight categories in two specific ethnic groups. While the study findings could not be extrapolated to the general population, they are very relevant locally due to the study’s national coverage. Moreover, they might be relevant to some populations in other countries with similar genetic and cultural characteristics, i.e., Arab populations in Western countries.

The observational nature of the study does not permit causal inferences.

Future studies that incorporate individual-level behavioral and environmental covariates could help address the limitations of the present study.

## 5. Conclusions

The findings of the present study show that many adolescents in Israel, even those who are not obese at ages 17–19, have an increasing BMI trajectory and move to a higher weight category in adulthood. In contrast, transition to lower weight categories is rare. This trend is especially prominent for Arab adolescents. Thus, given the steeper trend of BMI increase in the Arab population and the higher proportion of Jewish adolescents among all adolescents, public health efforts should focus on obesity prevention initiatives for the entire adolescent population. Furthermore, our study made it possible to pinpoint characteristics of adolescents that pose an especially high risk for further BMI increase, such as female gender, belonging to middle and low SES classes, and being an Arab male in the ‘class 2 obesity category’. These findings have the potential to guide the design of prevention programs in other countries with comparable population structures.

## Data Availability

The data that support the findings of this study are available on request from the corresponding author. The data are not publicly available due to privacy or ethical restrictions.

## Abbreviations

The following abbreviations are used in this manuscript:

BMI	Body mass index
SES	Socio-economic status
CHS	‘Clalit Health Services’
CDC	U.S. Centers for Disease Control and Prevention

## Appendix A

**Table A1:** Description and rationale for sensitivity analyses.

Rationale	Details
‘Missing at random’ assumption testing	Inverse probability weighting. Study participants were assigned weights based on the inverse probability of selection into the study, thus creating a pseudo-population that better represented the target population. These weights were derived from a logistic regression model that predicted the probability of being selected based on ethnicity, SES, and gender.
Different observation density in the two ethnic groups	Analyses limited to the first four measurement points per person
Possibility of a plateau/reversal in BMI in the late 20s.	Multilevel linear spline models. Knots at each of the ages 24–27 were tested, with the only significant knot at age 25.
Testing of the potential bias due to observation duration and attrition rate	Sensitivity analysis was conducted with an averaged BMI from a fixed age window (24–26 years)
Risk of residual bias due to the effects of regions/districts	An additional model with fixed effects for districts
Possible artifacts of categorical transitions around 18.5/25/30	Analysis with BMI as a continuous variable for the whole population of participants

SES = socio-economic status.

**Table A2:** Comparison of participants with and without BMI measurements.

Variable	Participants with Measurements (N = 101,679)	Participants Without Measurements or with Outlying Measurements (N = 157,566)	
Sex (male), N (%)	47,840 (47.0)	81,578 (51.8)	<0.001
Ethnicity (Jews), N (%)	48,363 (47.6)	84,100 (53.4)	<0.001
SES, N (%):			<0.001
low	37,163 (36.5)	49,683 (31.5)	
medium	46,986 (46.2)	77,278 (49.0)	
high	9145 (9.0)	18,493 (11.7)	
no data	8385 (8.2)	12,112 (7.7)	
District of residency, N (%): Northern Haifa Sharon-Shomron Central Dan-Petah-Tikva Jerusalem Southern No data	17,313 (17.0) 20,328 (20.0) 14,850 (14.6) 14,468 (14.2) 7121 (7.0) 12,408 (12.2) 15,166 (14.9) 25 (0.0)	26,348 (16.7) 30,489 (19.3) 21,492 (13.6) 22,290 (14.1) 11,121 (7.1) 23,331 (14.8) 22,444 (14.2) 51 (0.0)	<0.001

SES = socio-economic status.

**Table A3:** Baseline characteristics of adolescents.

Variable	CDC BMI Category											
	Underweight		Normal		Overweight		Obesity		Class 2 Obesity		Class 3 Obesity	
	Arabs (1861)	Jews (3313)	Arabs (38,209)	Jews (32,479)	Arabs (6167)	Jews (4940)	Arabs (5053)	Jews (5099)	Arabs (822)	Jews (999)	Arabs (319)	Jews (480)
Sex (male), N (%)	997 (53.6)	1779 (53.7)	17,647 ** (46.2)	14,662 (45.1)	2892 (46.9)	2281 (46.2)	2684 (53.1)	2679 (52.5)	404 (49.1)	514 (51.4)	169 (53.0)	235 (49.0)
SES, N (%) High Middle Low missing	17 *** (0.9) 487 (26.2) 1108 (59.5) 249 (13.4)	526 (15.9) 2324 (70.1) 318 (9.6) 145 (4.4)	277 *** (0.7) 9759 (25.5) 23,711 (62.1) 4462 (11.7)	6244 (19.2) 22,243 (68.5) 2565 (7.9) 1426 (4.4)	33 *** (5.3) 1515 (24.6) 3886 (63.0) 733 (11.8)	832 (16.8) 3466 (70.2) 409 (8.3) 233 (4.7)	21 *** (0.4) 1309 (25.9) 3160 (62.5) 563 (11.1)	801 (15.7) 3599 (70.6) 485 (9.5) 214 (4.2)	6 *** (0.7) 209 (25.4) 516 (62.8) 91 (11.1)	119 (11.9) 692 (69.3) 144 (14.4) 44 (4.4)	0 *** (0.0) 82 (25.7) 202 (63.3) 35 (11.0)	67 (14.0) 339 (70.6) 51 (10.6) 23 (4.8)
District of residence in Israel, N (%) Central Northern Haifa Sharon-Shomron Dan-PT Jerusalem Southern Missing	51 *** (2.7) 399 (21.4) 461 (24.8) 291 (15.6) 51 (2.7) 218 (11.7) 390 (21.0) 0 (0.0)	1045 (31.5) 331 (10.0) 467 (14.1) 364 (11.0) 409 (12.3) 181 (5.5) 506 (15.3) 0 (0.0)	641 *** (1.7) 8281 (21.7) 9277 (24.3) 6339 (16.6) 1209 (3.2) 6440 (16.8) 6022 (15.8) 1 (0.0)	9125 (28.1) 4196 (12.9) 5048 (15.5) 3883 (12.0) 3660 (11.3) 2039 (6.3) 4504 (13.9) 24 (0.1)	120 *** (1.9) 1182 (19.1) 1479 (24.0) 1055 (17.1) 208 (3.4) 1264 (20.5) 859 (13.9) 0 (0.0)	1262 (25.5) 705 (14.3) 744 (15.1) 632 (12.8) 535 (10.8) 339 (6.9) 722 (14.6) 1 (0.0)	87 *** (1.7) 920 (18.2) 1257 (24.9) 1000 (19.8) 179 (3.5) 1006 (19.9) 604 (11.9) 0 (0.0)	1424 (27.9) 599 (11.7) 753 (14.8) 611 (12.0) 538 (10.5) 763 (15.0) 811 (15.9) 0 (0.0)	20 *** (2.4) 142 (17.3) 188 (22.9) 176 (21.4) 14 (1.7) 193 (23.5) 89 (10.8) 0 (0.0)	255 (25.5) 137 (13.7) 146 (14.6) 117 (11.7) 85 (8.5) 87 (8.7) 171 (17.1) 1 (0.1)	10 *** (3.1) 43 (13.5) 81 (25.4) 62 (19.4) 11 (3.4) 76 (23.8) 36 (11.3) 0 (0.0)	118 (24.6) 52 (10.8) 71 (14.8) 51 (10.6) 72 (15.0) 34 (7.1) 82 (17.1) 0 (0.0)
Age of BMI measurement, years: Mean (SD) Median (IQR)	18.1 *** (0.8) 18.0 (17.4–18.7)	17.9 (0.7) 17.8 (17.3–18.2)	18.3 *** (0.9) 18.1 (17.3–18.8)	18.0 (0.8) 17.8 (17.3–18.3)	18.3 *** (0.9) 18.1 (17.3–18.8)	18.0 (0.8) 17.8 (17.2–18.4)	18.1 *** (0.7) 17.9 (17.3–18.5)	17.8 (0.7) 17.7 (17.2–18.2)	18.3 *** (0.9) 18.2 (17.5–19.0)	18.0 (0.8) 17.9 (17.2–18.6)	18.3 *** (0.9) 18.4 (17.6–19.2)	18.1 (0.8) 18.0 (16.3–18.6)
BMI, kg/m^2^ Mean (SD) Median (IQR)	16.9 *** (1.0) 16.9 (16.3–17.5)	16.7 (0.9) 16.8 (16.2–17.3)	21.5 *** (2.0) 21.5 (20.0–23.1)	21.0 (2.0) 20.9 (19.3–22.4)	26.6 *** (1.3) 26.4 (25.6–27.1)	26.4 (1.2) 26.2 (25.4–26.9)	30.4 (2.0) 30.1 (28.5–31.7)	30.5 (2.1) 30.1 (28.4–31.8)	36.8 (1.3) 36.6 (35.5–37.7)	37.0 * (1.4) 36.7 (35.5–37.8)	44.6 (4.6) 43.1 (40.7–45.4)	44.4 (4.4) 43.1 (40.7–45.5)
BMI percentile Mean (SD) Median (IQR)	2.5 *** (1.4) 2.8 (1.7–3.8)	2.2 (1.5) 2.2 (1.0–3.5)	45.1 *** (23.4) 48.4 (28.0–68.7)	42.0 (23.7) 41.1 (21.0–61.2)	89.6 *** (3.0) 90.0 (87.5–92.5)	90.0 (3.0) 90 (89.3–90.8)	97.7 *** (1.5) 97.8 (96.5–99.1)	97.9 (1.5) 98.2 (97.0–99.5)	99.1 *** (1.3) 99.9 (98.4–101.3)	99.5 (1.0) 99.9 (99.8–99.9)	99.2 *** (1.3) 100.0 (98.5–101.5)	99.5 (1.1) 100.0 (100.0–100.0)

Underweight = BMI < 5th percentile, normal weight = BMI 5th–84.9th percentile, overweight = BMI 85th–94.9th percentile, obesity = BMI ≥ 95th percentile, not including class 2 and class 3 obesity, class 2 obesity = BMI ≥ 120% to <140% of the 95th percentile or BMI ≥ 35 to <40 kg/m^2^, class 3 obesity = BMI ≥ 140% of the 95th percentile or BMI ≥ 40 kg/m^2^. SD = standard deviation, IQR = interquartile range. *** The differences are significant at *p* < 0.001, ** the differences are significant at *p* < 0.01, * the differences are significant at *p* < 0.05. SES = socio-economic status.

**Table A4:** Details of BMI measurements in young adulthood (ages 20 to <30 years).

	Arabs	Jews	*p*	Total
Participants, N (%)	52,431 (52.6)	47,310 (47.4)		99,741
BMI measurements, N (%)	231,072 (49.5)	236,120 (50.5)	<0.001	467,192
Mean (SD)	4.4 (3.7)	5.0 (4.9)		4.7 (4.4)
Median (IQR)	3 (2–5)	4 (2–6)		4 (2–6)
Participants (N (%)) with:			<0.001	
1 measurement	4950 (9.4)	5207 (11.0)		10,157 (10.2)
2 measurements	10,905 (20.8)	9165 (19.4)		20,070 (20.1)
3 measurements	11,243 (21.4)	8365 (17.7)		19,608 (19.7)
4 measurements	8130 (15.5)	6249 (13.2)		14,379 (14.4)
5 measurements	5218 (10.0)	4604 (9.7)		9822 (9.8)
6 measurements	3403 (6.5)	3300 (7.0)		6703 (6.7)
7 measurements	2248 (4.3)	2267 (4.8)		4515 (4.5)
8 measurements	1587 (3.0)	1802 (3.8)		3389 (3.4)
9 measurements	1084 (2.1)	1325 (2.8)		2409 (2.4)
10+ measurements	3663 (7.0)	5026 (10.6)		8689 (8.7)
Age of BMI measurement per participant, Mean (SD)	23.5 (2.8)	24.1 (2.9)	<0.001	23.8 (2.9)
Last age of BMI measurement per participant, Mean (SD)	25.9 (2.5)	26.4 (2.6)	<0.001	26.2 (2.5)
Measurement points per age, N (%)			<0.001	
20 years	49,678 (21.5)	29,942 (12.7)		79,620 (17.0)
21 years	26,418 (11.4)	27,425 (11.6)		53,844 (11.5)
22 years	23,008 (10.0)	26,432 (11.2)		49,441 (10.6)
23 years	21,107 (9.1)	23,875 (10.1)		44,982 (9.6)
24 years	21,590 (9.3)	22,601 (9.6)		44,191 (9.5)
25 years	25,163 (10.9)	22,426 (9.5)		47,589 (10.2)
26 years	22,844 (9.9)	22,275 (9.4)		45,119 (9.7)
27 years	18,459 (8.0)	21,239 (9.0)		39,698 (8.5)
28 years	13,687 (5.9)	20,251 (8.6)		33,939 (7.3)
29 years	9118 (3.9)	19,654 (8.3)		28,772 (6.2)

SD = standard deviation, IQR = interquartile range.

**Table A5:** Transition to a higher BMI category from adolescence to young adulthood.

	Underweight ***		Normal ***		Overweight ***		Obesity	
	Arabs (1861)	Jews (3313)	Arabs (38,209)	Jews (32,478)	Arabs (6167)	Jews (4940)	Arabs (5053)	Jews (5099)
N (%)	1295 (69.6)	1974 (59.6)	13,188 (34.5)	8552 (26.3)	2150 (34.9)	1534 (31.1)	1253 (24.8)	1250 (24.5)

Underweight = BMI < 5th percentile, normal weight = BMI 5th–84.9th percentile, overweight = BMI 85th = 94.9th percentile, obesity = BMI ≥ 95th percentile, not including class 2 and class 3 obesity. *** The differences are significant at *p* < 0.001.

**Table A6:** Summary of results for the four nested multilevel models estimating BMI trajectory for each adolescent weight group.

	Null Model	Model 1	Model 2	Model 3 (Final)
‘Underweight’ weight category in adolescence				
Regression coefficients, fixed effects (Standard errors)				
Intercept	19.06 (0.03) ***	17.94 (0.04) ***	17.89 (0.13) ***	17.79 (0.14) ***
Age		0.25 (0.01) ***	0.25 (0.01) ***	0.27 (0.01) ***
Ethnicity (Jews)			−0.78 (0.08) ***	−0.63 (0.10) ***
Sex (Male)			1.03 (0.06) ***	1.04 (0.06) ***
SES				
High (reference)				
Middle			0.00 (0.11)	0.00 (0.11)
Low			−0.01 (0.13)	−0.02 (0.13)
Age × Ethnicity (Jews)				−0.04 (0.02) *
Variance components, random effects (Standard deviations)				
Within person	4.60 (2.14)	6.48 (2.54)	5.98 (2.45)	5.98 (2.44)
Between person	4.33 (2.08)	2.87 (1.69)	2.87 (1.69)	2.87 (1.69)
Time trajectory (age in adulthood)		0.13 (0.37)	0.13 (0.37)	0.14 (0.37)
Model summary				
AIC	108,321.9	104,169.3	103,789.5	103,780.5
BIC	108,346.1	104,217.6	103,878.2	103.853.0
Deviance	108,315.9	104.157.3	103,767.5	103.762.5
‘Normal’ weight category in adolescence				
Regression coefficients, fixed effects (Standard errors)				
Intercept	23.07 (0.01) ***	21.80 (0.01) ***	21.39 (0.05) ***	21.30 (0.05) ***
Age		0.28 (0.00) ***	0.28 (0.00) ***	0.31 (0.00) ***
Ethnicity (Jews)			−0.67 (0.03) ***	−0.46 (0.03) ***
Sex (Male)			0.81 (0.02) ***	0.82 (0.01) ***
SES				
High (reference)				
Middle			0.35 (0.04) ***	0.35 (0.04) ***
Low			0.39 (0.06) ***	0.42 (0.05) ***
Age × Ethnicity (Jews)				−0.06 (0.00) ***
Variance components, random effects (Standard deviations)				
Within person	8.34 (2.89)	8.27 (2.88)	7.95 (2.82)	7.95 (2.81)
Between person	5.97 (2.44)	3.96 (1.99)	3.96 (2.00)	3.96 (2.00)
Time trajectory (age in adulthood)		0.15 (0.39)	0.15 (0.39)	0.15 (0.39)
Model summary				
AIC	1,516,453.8	1,457,360.9	1,454,816.3	1,454,628.5
BIC	1,516,485.7	1,457,424.6	1,454,933.1	1,454,755.8
Deviance	1,516,447.8	1,457,348.9	1,454,794.3	1,454,604.5
‘Overweight’ weight category in adolescence				
Regression coefficients, fixed effects (Standard errors)				
Intercept	27.91 (0.03) ***	26.38 (0.03) ***	25.82 (0.14) ***	25.74 (0.14) ***
Age		0.32 (0.01) ***	0.32 (0.01) ***	0.35 (0.01) ***
Ethnicity (Jews)			−0.25 (0.08) **	−0.05 (0.09)
Sex (Male)			−0.25 (0.06) ***	−0.24 (0.06) ***
SES				
High (reference)				
Middle			0.77(0.12) ***	0.77 (0.12) ***
Low			0.85 (0.14) ***	0.84 (0.14) ***
Age × Ethnicity (Jews)				−0.07 (0.01) ***
Variance components, random effects (Standard deviations)				
Within person	10.60 3.26	8.55 (2.92)	8.57 (2.92)	8.56 (2.93)
Between person	8.64 2.94	5.43 (2.33)	5.43 (2.33)	5.43 (2.33)
Time trajectory (age in adulthood)		0.27 (0.52)	0.27 (0.52)	0.27 (0.52)
Model summary				
AIC	304,255.6	289,142.9	289,042.0	289,019.8
BIC	304,282.4	289,196.6	289,140.5	289,127.2
Deviance	304.249.6	289.130.9	289,020.0	288,995.8
***‘Obesity’ weight category in adolescence***				
Regression coefficients, fixed effects (Standard errors)				
Intercept	31.31 (0.04) ***	30.12 (0.05) ***	29.42 (0.19) ***	29.22 (0.19) ***
Age		0.23 (0.01) ***	0.23 (0.01) ***	0.29 (0.01) ***
Ethnicity (Jews)			0.18 (0.10)	0.60 (0.12) ***
Sex (Male)			−0.83 (0.08) ***	−0.82 (0.08) ***
SES				
High (reference)				
Middle			0.89 (0.16) ***	0.88 (0.16) ***
Low			1.26 (0.22) ***	1.25 (0.22) ***
Age × Ethnicity (Jews)				−0.12 (0.02) ***
Variance components, random effects (Standard deviations)				
Within person	15.91 (3.99)	17.69 (4.21)	17.69 (4.21)	17.64 (4.20)
Between person	13.00 (3.61)	8.81 (2.97)	8.81 (2.97)	8.81 (2.97)
Time trajectory (age in adulthood)		0.47 (0.69)	0.47 (0.69)	0.47 (0.69)
Model summary				
AIC	361,153.9	348,354.9	348,191.1	348,140.6
BIC	361,181.1	348,409.2	348,290.7	348,259.2
Deviance	361,147.9	348,342.9	348,169.1	348,126.6
***‘Class 2 obesity’ weight category in adolescence***				
Regression coefficients, fixed effects (Standard errors)				
Intercept	35.90 (0.12)	36.09 (0.16) ***	34.89 (0.52) ***	34.61(0.53) ***
Age		−0.09 (0.03) **	−0.09 (0.03) **	−0.01 (0.05)
Ethnicity (Jews)			−0.05 (0.27)	0.46 (0.35)
Sex (Male)			−0.31 (0.23)	−0.30 (0.23)
SES				
High (reference)				
Middle			1.45 (0.46) **	1.45 (0.46) **
Low			1.51 (0.51) **	1.50 (0.51) **
Age × Ethnicity (Jews)				−0.14 (0.06) *
Variance components, random effects (Standard deviations)				
Within person	19.63 (4.43)	30.09 (5.49)	30.03 (5.48)	29.98 (5.48)
Between person	21.82 (4.67)	15.26 (3.91)	15.26 (3.91)	15.26 (3.91)
Time trajectory (age in adulthood)		1.04 (1.02)	1.04 (1.02)	1.04 (1.02)
Model summary				
AIC	91,617.0	88,981.3	88,978.2	88,974.8
BIC	91,639.9	89,027.0	89,061.9	89,066.1
Deviance	91,611.0	88,969.3	88,956.2	88,950.8
***‘Class 3 obesity’ weight category in adolescence***				
Regression coefficients, fixed effects (Standard errors)				
Intercept	38.60 (0.25) ***	40.18 (0.34) ***	38.98 (1.15) ***	38.83 (1.19) ***
Age		−0.41 (0.05) ***	−0.41 (0.05) ***	−0.37 (0.09) ***
Ethnicity (Jews)			0.78 (0.66)	1.01 (0.79)
Sex (Male)			0.23 (0.51)	0.23 (0.51)
SES				
High (reference)				
Middle			0.17 (0.96)	0.17 (0.96)
Low			1.64 (1.13)	1.64 (1.13)
Age × Ethnicity (Jews)				−0.06 (0.11)
Variance components, random effects (Standard deviations)				
Within person	44.58 (6.68)	71.73 (8.47)	71.67 (8.47)	71.65 (8.47)
Between person	31.13 (5.58)	19.99 (4.47)	19.99 (4.47)	19.99 (4.47)
Time trajectory (age in adulthood)		1.64 (1.28)	1.64 (1.28)	1.64 (1.28)
Model summary				
AIC	52,258.5	50,221.4	50,226.4	50,228.1
BIC	52,279.5	50,263.4	50,303.3	50,312.0
Deviance	52,252.5	50,209.4	50,204.4	50,204.1

Underweight = BMI < 5th percentile, normal weight = BMI 5th–84.9th percentile, overweight = BMI 85th–94.9th percentile, obesity = BMI ≥ 95th percentile, not including class 2 and class 3 obesity, class 2 obesity = BMI ≥ 120% to <140% of the 95th percentile or BMI ≥ 35 to <40 kg/m^2^, class 3 obesity = BMI ≥ 140% of the 95th percentile or BMI ≥ 40 kg/m^2^. *** The differences are significant at *p* < 0.001, ** the differences are significant at *p* < 0.01, * the differences are significant at *p* < 0.05. SES = socio-economic status. AIC = Akaike information criterion, BIC = Bayesian information criterion.

**Table A7:** Cumulative difference in predicted BMI between the two ethnic groups after 10 years of follow-up.

	Underweight	Normal Weight	Overweight	Obesity	Class 2 Obesity	Class 3 Obesity
Difference in predicted BMI (95% CI)	1.02 (0.77; 1.27)	1.09 (1.00; 1.17)	0.74 (0.49; 0.99)	0.61 (0.30; 0.92)	0.95 (0.02; 1.98)	−0.42 (−2.27; 1.43)
*p*-value	<0.001	<0.001	<0.001	<0.001	0.045	0.656

Underweight = BMI < 5th percentile, normal weight = BMI 5th–84.9th percentile, overweight = BMI 85th–94.9th percentile, obesity = BMI ≥ 95th percentile, not including class 2 and class 3 obesity, class 2 obesity = BMI ≥ 120% to <140% of the 95th percentile or BMI ≥ 35 to <40 kg/m^2^, class 3 obesity = BMI ≥ 140% of the 95th percentile or BMI ≥ 40 kg/m^2^.

**Table A8:** Summary of results of the sensitivity analysis on a pseudo-population, estimating the BMI trajectory for each adolescent weight group using inverse probability weighting.

	Underweight	Normal Weight	Overweight	Obesity	Class 2 Obesity	Class 3 Obesity
Regression coefficients, fixed effects (Standard errors)						
Intercept	17.80 (0.14) ***	21.31 (0.05) ***	25.75 (0.14) ***	29.22 (0.19) ***	34.59(0.53) ***	38.80 (1.19) ***
Age	0.27 (0.01) ***	0.31 (0.00) ***	0.35 (0.01) ***	0.29 (0.01) ***	−0.01 (0.05)	−0.37 (0.09) ***
Ethnicity (Jews)	−0.62 (0.10) ***	−0.45 (0.03) ***	−0.04 (0.09)	0.60 (0.12) ***	0.46 (0.35)	1.05 (0.80)
Sex (Male)	1.04 (0.06) ***	0.82 (0.02) ***	−0.25 (0.06) ***	−0.83 (0.08) ***	−0.31 (0.23)	0.23 (0.51)
SES High (reference) Middle Low	0.00 (0.10) 0.02 (0.13)	0.35 (0.04) *** 0.42(0.05) ***	0.77 (0.12) *** 0.84 (0.14) ***	0.89 (0.16)*** 1.38 (0.18)***	1.47 (0.46) ** 1.54 (0.51) **	0.18 (0.96) 1.67 (1.12)
Age × Ethnicity (Jews)	−0.04 (0.02) *	−0.06 (0.00) ***	−0.07 (0.01) ***	−0.12 (0.02) ***	−0.14 (0.06) *	−0.07 (0.11)
Variance components, random effects (Standard deviations)						
Within person	6.09 (2.47)	7.97 (2.82)	8.56 (2.92)	17.73 (4.21)	30.34 (5.51)	71.81 (8.47)
Between person	7.06 (2.66)	9.53 (3.09)	13.02 (3.61)	21.57 (4.64)	37.72 (6.14)	49.86 (7.06)
Time trajectory (age in adulthood)	0.14 (0.37)	0.15 (0.39)	0.27 (0.52)	0.47 (0.69)	1.04 (1.02)	1.64 (1.28)

Underweight = BMI < 5th percentile, normal weight = BMI 5th–84.9th percentile, overweight = BMI 85th–94.9th percentile, obesity = BMI ≥ 95th percentile, not including class 2 and class 3 obesity, class 2 obesity = BMI ≥ 120% to <140% of the 95th percentile or BMI ≥ 35 to <40 kg/m^2^, class 3 obesity = BMI ≥ 140% of the 95th percentile or BMI ≥ 40 kg/m^2^. *** The differences are significant at *p* < 0.001, ** the differences are significant at *p* < 0.01, * the differences are significant at *p* < 0.05. SES = socio-economic status.

**Table A9:** Summary of the results of the sensitivity analysis estimating BMI trajectory for each adolescent weight group using the first four measurement points per person.

	Underweight	Normal Weight	Overweight	Obesity	Class 2 Obesity	Class 3 Obesity
Regression coefficients, fixed effects (Standard errors)						
Intercept	17.80 (0.14) ***	21.33 (0.05) ***	25.73 (0.14) ***	29.25 (0.19) ***	34.78 (0.54) ***	38.75 (1.20) ***
Age	0.27 (0.01) ***	0.30 (0.00) ***	0.35 (0.01) ***	0.30 (0.01) ***	0.06 (0.05)	−0.38 (0.10) ***
Ethnicity (Jews)	−0.60 (0.11) ***	−0.41 (0.03) ***	−0.04 (0.09)	0.47 (0.12) ***	0.22 (0.34)	0.50 (0.78)
Sex (Male)	1.05 (0.06) ***	0.83 (0.02) ***	−0.16 (0.06) ***	−0.72 (0.08) ***	−0.34 (0.23)	0.23 (0.53)
SES High (reference) Middle Low	0.00 (0.11) 0.02 (0.13)	0.34 (0.04) *** 0.40 (0.05) ***	0.72 (0.12) *** 0.79 (0.14) ***	0.80 (0.16) *** 1.22 (0.18) ***	1.17 (0.46) ** 1.06 (0.52) **	0.36 (0.99) 1.55 (1.16)
Age × Ethnicity (Jews)	−0.04 (0.02) *	−0.08 (0.00) ***	−0.07 (0.02) ***	−0.11 (0.02) ***	−0.12 (0.07)	−0.07 (0.14)
Variance components, random effects (Standard deviations)						
Within person	6.09 (2.47)	7.97 (2.82)	8.56 (2.92)	17.73 (4.21)	30.34 (5.51)	71.81 (8.47)
Between person	7.06 (2.66)	9.53 (3.09)	13.02 (3.61)	21.57 (4.64)	37.72 (6.14)	49.86 (7.06)
Time trajectory (age in adulthood)	0.14 (0.37)	0.15 (0.39)	0.27 (0.52)	0.47 (0.69)	1.04 (1.02)	1.64 (1.28)

Underweight = BMI < 5th percentile, normal weight = BMI 5th–84.9th percentile, overweight = BMI 85th–94.9th percentile, obesity = BMI ≥ 95th percentile, not including class 2 and class 3 obesity, class 2 obesity = BMI ≥ 120% to <140% of the 95th percentile or BMI ≥ 35 to <40 kg/m^2^, class 3 obesity = BMI ≥ 140% of the 95th percentile or BMI ≥ 40 kg/m^2^. *** The differences are significant at *p* < 0.001, ** the differences are significant at *p* < 0.01, * the differences are significant at *p* < 0.05. SES = socio-economic status.

**Table A10:** Sensitivity analysis for change in BMI category from adolescence to young adulthood with an averaged BMI from 24 to 26 years as the outcome.

	Weight Category in Adolescence											
Weight Category in Adulthood	Underweight		Normal		Overweight		Obesity		Class 2 Obesity		Class 3 Obesity	
	Arabs (N = 2260)	Jews (N = 43,830)	Arabs (N = 46,388)	Jews (N = 41,025)	Arabs (N = 9228)	Jews (N = 7664)	Arabs (N = 8905)	Jews (N = 9964)	Arabs (N = 1858)	Jews (N = 2687)	Arabs (N = 958)	Jews (N = 1579)
Underweight (N, %)	800 (4.0)	2244 (7.4)	1839 (4.0)	3032 (7.4)	20 (0.2)	27 (0.4)	5 (0.1)	13 (0.1)	2 (0.1)	0 (0.0)	0 (0.0)	3 (0.2)
Normal (N, %)	1351 (59.0)	2043 (66.8)	27,384 (59.0)	27,421 (66.8)	1376 (14.9)	1720 (22.4)	547 (6.1)	932 (9.4)	85 (4.6)	147 (5.5)	35 (3.7)	63 (4.0)
Overweight (N, %)	96 (28.1)	84 (20.6)	13,034 (28.1)	8440 (20.6)	3771 (40.9)	3266 (42.6)	2171 (24.4)	2632 (26.4)	223 (12.0)	391 (14.6)	140 (14.6)	178 (11.3)
Obesity (N, %)	11 (6.7)	8 (4.3)	3100 (6.7)	1771 (4.3)	2779 (30.1)	1871 (24.4)	3058 (34.3)	3101 (31.1)	371 (20.0)	627 (23.3)	154 (16.1)	283 (17.9)
Obesity class 2 (N, %)	1 (1.7)	1 (0.7)	784 (1.7)	272 (0.7)	1012 (11.0)	615 (6.0)	2011 (22.6)	2252 (22.6)	527 (28.4)	756 (28.1)	177 (18.5)	277 (17.5)
Obesity class 3 (N, %)	1 (0.5)	3 (0.2)	247 (0.5)	89 (0.2)	270 (2.9)	165 (2.2)	1113 (12.5)	1034 (10.4)	650 (35.0)	766 (28.5)	152 (47.2)	775 (49.1)
*p*-value (Chi-squared test)	<0.001		<0.001		<0.001		<0.001		<0.001		0.10	

Underweight = BMI < 5th percentile, normal weight = BMI 5th–84.9th percentile, overweight = BMI 85th–94.9th percentile, obesity = BMI ≥ 95th percentile, not including class 2 and class 3 obesity, class 2 obesity = BMI ≥ 120% to <140% of the 95th percentile or BMI ≥ 35 to <40 kg/m^2^, class 3 obesity = BMI ≥ 140% of the 95th percentile or BMI ≥ 40 kg/m^2^.

**Table A11:** Summary of the results for the spline linear multilevel models estimating BMI trajectory for each adolescent weight group.

	Underweight	Normal Weight	Overweight	Obesity	Class 2 Obesity	Class 3 Obesity
Regression coefficients, fixed effects (Standard errors)						
Intercept	17.86 (0.14) ***	21.33 (0.05) ***	25.71 (0.14) ***	29.5 (0.19) ***	34.71 (0.54) ***	38.85 (1.19) ***
Age	0.25 (0.01) ***	0.30 (0.00) ***	0.36 (0.01) ***	0.32 (0.01) ***	−0.04 (0.05)	−0.37 (0.09) ***
Ethnicity (Jews)	−0.59 (0.10) ***	−0.44 (0.03) ***	−0.07 (0.09)	−0.56 (0.12) ***	0.51 (0.35)	1.02 (0.79)
Sex (Male)	1.04 (0.06) ***	0.82 (0.02) ***	−0.24 (0.06) ***	−0.82 (0.08) ***	−0.29 (0.23)	0.23 (0.51)
SES High (reference) Middle Low	0.00 (0.11) 0.02 (0.13)	0.35 (0.04) *** 0.40 (0.05) ***	0.77 (0.12) *** 0.84 (0.14) ***	0.88 (0.16) *** 1.37 (0.18) ***	1.44 (0.46) ** 1.50 (0.51) **	0.17 (0.96) 1.64 (1.13)
Age × Ethnicity (Jews)	−0.05 (0.02) **	−0.07 (0.00) ***	−0.07 (0.01) ***	−0.11 (0.02) ***	−0.15 (0.06) *	−0.06 (0.11)
Spline	0.10 (0.02) ***	0.04 (0.01) ***	−0.05 (0.02) **	0.09 (0.02) ***	0.12 (0.06) *	0.02 (0.09)
Variance components, random effects (Standard deviations)						
Within person	5.97 (2.44)	7.95 (2.82)	8.56 (2.93)	14.62 (3.82)	30.00 (5.48)	71.67 (8.47)
Between person	2.86 (1.69)	3.96 (1.99)	5.43 (2.33)	6.84 (2.62) (4.64)	15.26 (3.91)	19.99 (4.47)
Time trajectory (age in adulthood)	0.14 (0.37)	0.15 (0.39)	0.27 (0.52)	0.40 (0.63)	1.03 (1.02)	1.63 (1.28)
Model summary						
AIC	103,764.3	1,454,590.1	289,014.6	348,134.9	88,973.0	50,230.1
BIC	103,869.0	1,454,728.1	289,131.0	348,252.6	89,071.9	50,321.0
Deviance	103,738.3	1,454,564.1	288,988.6	348.108.9	88,947.0	50,204.1

Underweight = BMI < 5th percentile, normal weight = BMI 5th–84.9th percentile, overweight = BMI 85th–94.9th percentile, obesity = BMI ≥ 95th percentile, not including class 2 and class 3 obesity, class 2 obesity = BMI ≥ 120% to <140% of the 95th percentile or BMI ≥ 35 to <40 kg/m^2^, class 3 obesity = BMI ≥ 140% of the 95th percentile or BMI ≥ 40 kg/m^2^. *** The differences are significant at *p* < 0.001, ** the differences are significant at *p* < 0.01, * the differences are significant at *p* < 0.05. SES = socio-economic status. AIC = Akaike information criterion, BIC = Bayesian information criterion.

**Table A12:** Summary of models’ fit assessment from the sensitivity analysis estimating BMI trajectory for each adolescent weight group with an additional fixed effect for the district.

	Underweight	Normal Weight	Overweight	Obesity	Class 2 Obesity	Class 3 Obesity
AIC	103,792.1	1,454,602.2	288,995.0	348,121.9	88,984.8	50,219.2
BIC	103,953.1	1,454,835.7	289,183.0	348,303.0	89,144.6	50,359.0
Deviance	103,752.1	1,454,5582	288,953.0	348.081.9	88,942.8	50,179.2

Underweight = BMI < 5th percentile, normal weight = BMI 5th–84.9th percentile, overweight = BMI 85th–94.9th percentile, obesity = BMI ≥ 95th percentile, not including class 2 and class 3 obesity, class 2 obesity = BMI ≥ 120% to <140% of the 95th percentile or BMI ≥ 35 to <40 kg/m^2^, class 3 obesity = BMI ≥ 140% of the 95th percentile or BMI ≥ 40 kg/m^2^.

**Table A13:** Summary of results for the four nested multilevel models estimating BMI trajectory for the whole study cohort.

	Null Model	Model 1	Model 2	Model 3 (Final)
Regression coefficients, fixed effects (Standard errors)				
Intercept	24.33 (0.02) ***	23.37 (0.02) ***	22.43 (0.06) ***	22.3 (0.07) ***
Age		0.26 (0.00) ***	0.26 (0.00) ***	0.30 (0.00) ***
Ethnicity (Jews)			−0.38 (0.04) ***	−0.09 (0.04) *
Sex (Male)			1.03 (0.06) ***	1.04 (0.06) ***
SES				
High (reference)				
Middle			0.80 (0.06) ***	0.80 (0.06) ***
Low			1.03 (0.07) ***	1.03 (0.07) ***
Age × Ethnicity (Jews)				−0.08 (0.00) **
Variance components, random effects (Standard deviations)				
Within person	24.66 (4.97)	24.24 (4.92)	24.00 (4.90)	23.97 (4.90)
Between person	5.60 (2.37)	5.59 (2.36)	5.59 (2.36)	5.59 (2.36)
Time trajectory (age in adulthood)		0.24 (0.49)	0.24 (0.49)	0.24 (0.49)
Model summary				
AIC	2,481,287.0	2,469,381.0	2,468,241.0	2,467,966.0
BIC	2,481,342.0	2,469,447.0	2,468,372.0	2,468,099.0
Deviance	2,481,277.0	2,469,369.0	2,468,229.0	2,467,942.0

*** The differences are significant at *p* < 0.001, ** the differences are significant at *p* < 0.01, * the differences are significant at *p* < 0.05. SES = socio-economic status. AIC = Akaike information criterion, BIC = Bayesian information criterion.
